# The Effects of Apolipoprotein F Deficiency on High Density Lipoprotein Cholesterol Metabolism in Mice

**DOI:** 10.1371/journal.pone.0031616

**Published:** 2012-02-20

**Authors:** William R. Lagor, David W. Fields, Sumeet A. Khetarpal, Arthi Kumaravel, Wen Lin, Nathaniel Weintraub, Kaijin Wu, Sarah F. Hamm-Alvarez, Denise Drazul-Schrader, Margarita de la Llera-Moya, George H. Rothblat, Daniel J. Rader

**Affiliations:** 1 Division of Translational Medicine and Human Genetics, Institute for Translational Medicine and Therapeutics, Perelman School of Medicine, University of Pennsylvania, Philadelphia, Pennsylvania, United States of America; 2 Department of Pharmacology and Pharmaceutical Sciences, University of Southern California, Los Angeles, California, United States of America; 3 Division of Gastroenterology, Hepatology and Nutrition, The Children's Hospital of Philadelphia, Philadelphia, Pennsylvania, United States of America; University of Tor Vergata, Italy

## Abstract

Apolipoprotein F (apoF) is 29 kilodalton secreted sialoglycoprotein that resides on the HDL and LDL fractions of human plasma. Human ApoF is also known as Lipid Transfer Inhibitor protein (LTIP) based on its ability to inhibit cholesteryl ester transfer protein (CETP)-mediated transfer events between lipoproteins. In contrast to other apolipoproteins, ApoF is predicted to lack strong amphipathic alpha helices and its true physiological function remains unknown. We previously showed that overexpression of Apolipoprotein F in mice reduced HDL cholesterol levels by 20–25% by accelerating clearance from the circulation. In order to investigate the effect of physiological levels of ApoF expression on HDL cholesterol metabolism, we generated ApoF deficient mice. Unexpectedly, deletion of ApoF had no substantial impact on plasma lipid concentrations, HDL size, lipid or protein composition. Sex-specific differences were observed in hepatic cholesterol content as well as serum cholesterol efflux capacity. Female ApoF KO mice had increased liver cholesteryl ester content relative to wild type controls on a chow diet (KO: 3.4+/−0.9 mg/dl vs. WT: 1.2+/−0.3 mg/dl, p<0.05). No differences were observed in ABCG1-mediated cholesterol efflux capacity in either sex. Interestingly, ApoB-depleted serum from male KO mice was less effective at promoting ABCA1-mediated cholesterol efflux from J774 macrophages relative to WT controls.

## Introduction

Apolipoprotein F (apo F) is an HDL-associated protein that bears no structural or sequence similarity to the other classical apolipoproteins [1], [2]. This includes the absence of strong predicted amphipathic alpha helices which are essential for the lipid binding properties of other HDL-associated apolipoproteins such as apo A-I, apo A-II, apo E and the apo Cs [3], [4]. Apo F is a highly acidic secreted sialoglycoprotein [5] which was originally described as lipid transfer inhibitor protein (LTIP), based on its ability to inhibit cholesteryl ester transfer protein (CETP) activity *ex vivo* [*6]*, [7], [8], [9], [10**]. LDL-associated apo F has been shown to selectively inhibit CETP-mediated transfer events involving the LDL particle [11], [12]. The function of HDL-associated apo F, which represents greater than 75% of the total plasma pool [13], [14], is currently not known. The genes for Apolipoprotein A-I (ApoA-I) and ApoF have both been found in nearly every mammalian and fish species examined [15], [16], [17]. Likewise the CETP gene also exists in fish, but is absent in mice and rats [18] and functionally defective in several other mammals- pigs, cows, horses and dogs [19]. This lack of a consistent requirement for plasma CE transfer activity across species suggests that ApoF has been conserved throughout evolution for a purpose distinct from CETP inhibition.

Recent proteomics studies have identified over 50 unique proteins as *bona fide* components of HDL [20], [21], [22]. Surprisingly, many of these proteins have no known role in lipid transport, but instead are believed to participate in processes as diverse as protease inhibition, complement activation, inflammation, platelet function, and innate immunity [20]. It has been suggested that these proteins are organized into subclasses where an HDL particle may contain several related proteins that act cooperatively to perform their function [23], [24]. Interrogating the roles of these individual HDL proteins is an important endeavor if we are to understand the diverse functions of HDL, and its inverse relationship to cardiovascular disease. We previously showed that overexpression of both mouse and human apo F reduced HDL cholesterol levels by 20–25% in mice [14], suggesting that apo F may be an important new player in HDL metabolism. In addition, plasma from mice overexpressing apoF had an improved ability to accept cholesterol from the macrophage per HDL-C. To investigate the physiological relevance of these findings, we knocked out the ApoF gene in mice. We report that mice with targeted deletion of the ApoF gene are viable and fertile, and exhibit no major perturbations in HDL cholesterol concentrations, size, lipid or protein composition. The only lipid change observed was an increase in hepatic cholesterol content in the Female ApoF KO mice. Despite the lack of effects on HDL quantity, size and composition, we found that ApoB-depleted sera from the male ApoF KO mice had a reduced ability to promote cholesterol efflux via the ABCA1 transporter relative to WT controls.

## Results

**Figure 1:**
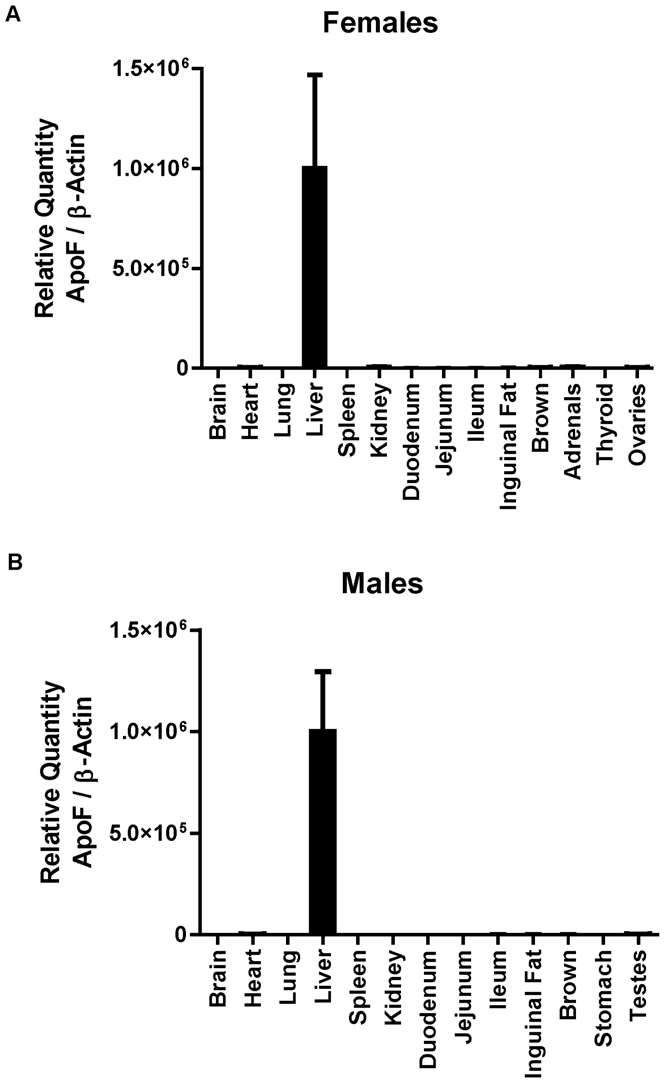
Tissue expression, processing and glycosylation of murine apolipoprotein F. *A.* Real time RT-PCR analysis of total RNA from tissues of female mice: brain, heart, lung, liver, spleen, kidney, duodenum, jejunum, ileum, inguinal fat, brown fat, adrenals, thyroid, and ovaries. *B.* Real time RT-PCR analysis of total RNA from tissues of male mice: brain, heart, lung, liver, spleen, kidney, duodenum, jejunum, ileum, inguinal fat, brown fat, stomach and testes. Values are shown as the mean +/− standard deviation for each animal (n = 3 per group).

Apo F is a secreted protein known to be highly expressed in hamster and human liver- two species with endogenous CETP expression. We used real time RT-PCR to evaluate the expression pattern of apo F in mice, a species without a functional CETP gene. We found that mouse ApoF is virtually exclusively liver-expressed, with message levels ranging from low to undetectable in the other whole tissues that were examined. (Figure 1A and 1B). In the liver, ApoF was detected at a threshold cycle (C_t_) of 21.9, appearing just before β-Actin (C_t_ = 22.2). ApoF was detectable in the testes (C_t_ = 30.9) adrenals (C_t_ = 31.5), and ovaries (C_t_ = 33.5) at levels less than 0.6% of that seen in the liver. (Standard curves and C_t_ values for all tissues are included in Figure S1, and Table S1 and Table S2.)

**Figure 2:**
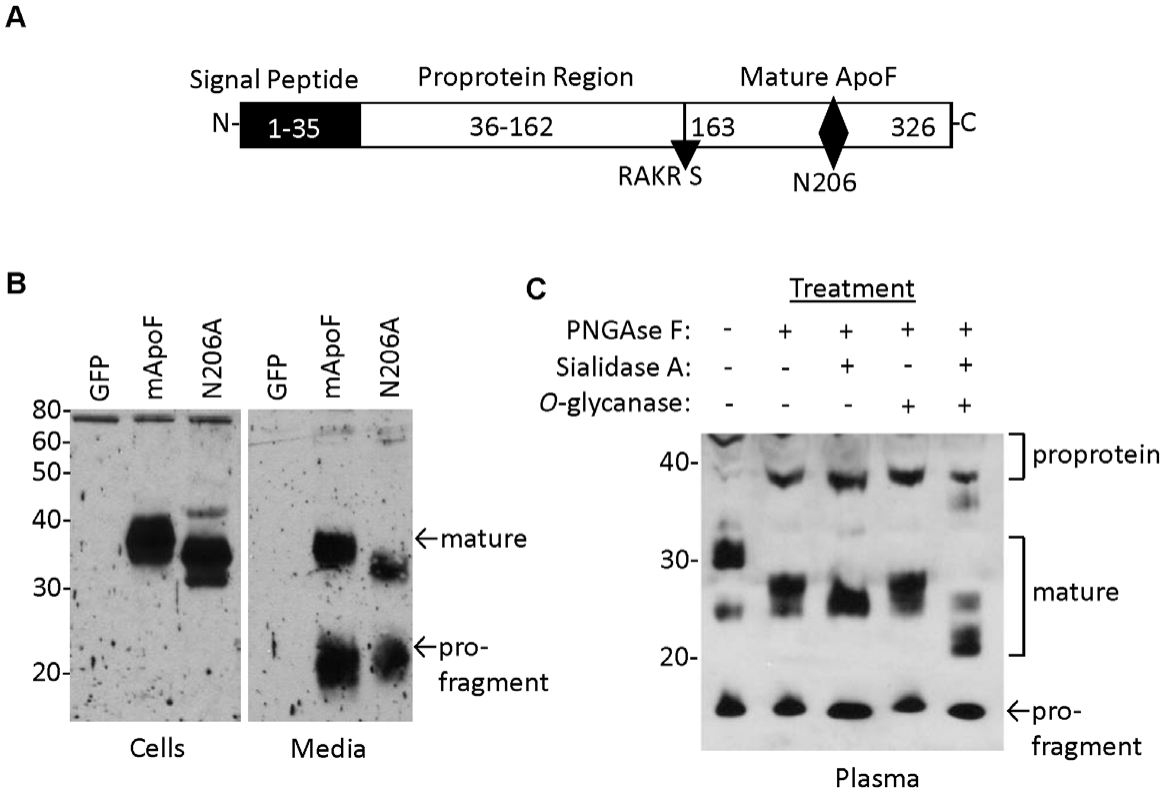
Glycosylation, processing and secretion of the mouse apo F protein. *A.* Schematic of the mouse apo F precursor protein depicting the predicted boundaries of the signal peptide, proprotein region, and furin cleavage site (RAKR/S). The N-linked glycosylation site at N206 is shown with a diamond. *B.* Western blot for mouse apo F in cells (left) and media (right), from HEK293 cells transiently transfected with either green fluorescent protein (GFP), wild type mouse ApoF (mApoF), or mouse apo F with asparagine 206 mutated to alanine (N206A). *C.* Western blot of apo F in one microliter of plasma from mice overexpressing mApoF from a liver-specific AAV vector. Plasma was denatured with heat and then subjected with deglycosylation by PNGase F, Sialidase A, and O-glycanase. The mature and pro-fragment portions of the apo F protein are shown with arrows.

**Figure 3:**
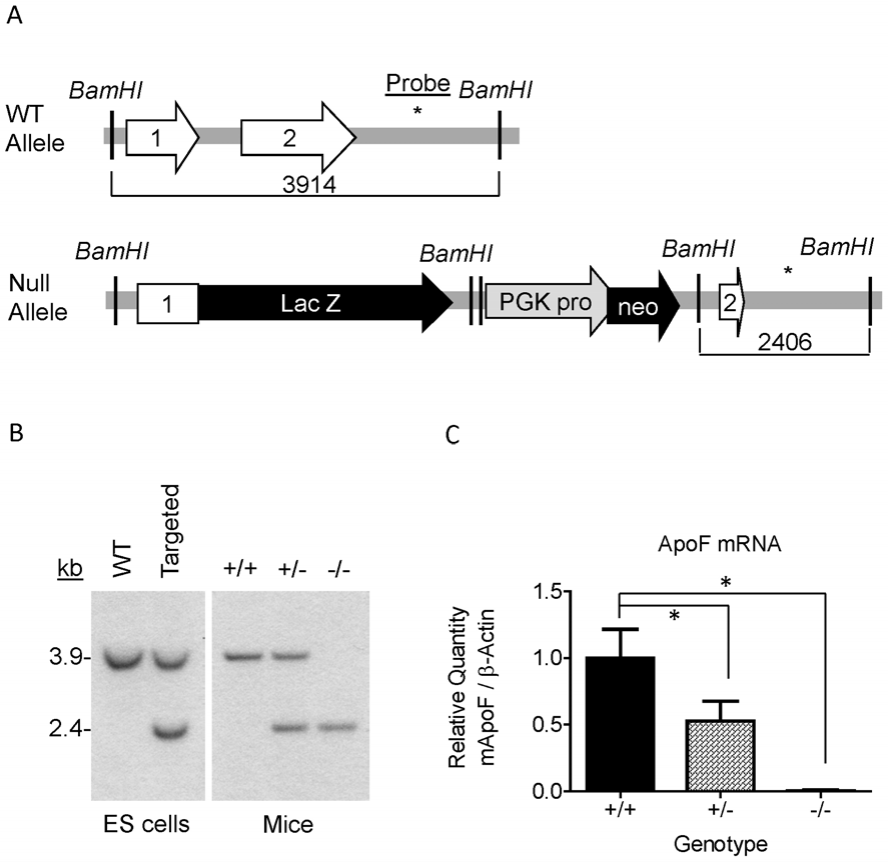
Generation of Apolipoprotein F Deficient Mice. *A.* Schematic diagram (not to scale) showing the wild type and deleted ApoF alleles. Features are depicted as follows: Exons- white arrows, Beta galactosidase reporter gene- “LacZ” black arrow, PGK promoter- grey arrow, Neomycin resistance gene- “Neo” black arrow, *BamHI* restriction sites- vertical lines, and the location of Southern Blotting probe- asterisk. *B.* Southern blot confirming successful targeting and genomic location of null allele: WT allele yields a 3.9 kb band, while the Null allele is 2.4 kb. The left panel contains ES cell DNA, while the right panel depicts livers from the mice. *C.* Real Time RT-PCR data on ApoF mRNA in the livers of female wild type (+/+), heterozygous (+/−), and homozygous (−/−) ApoF deficient mice.

**Figure 4:**
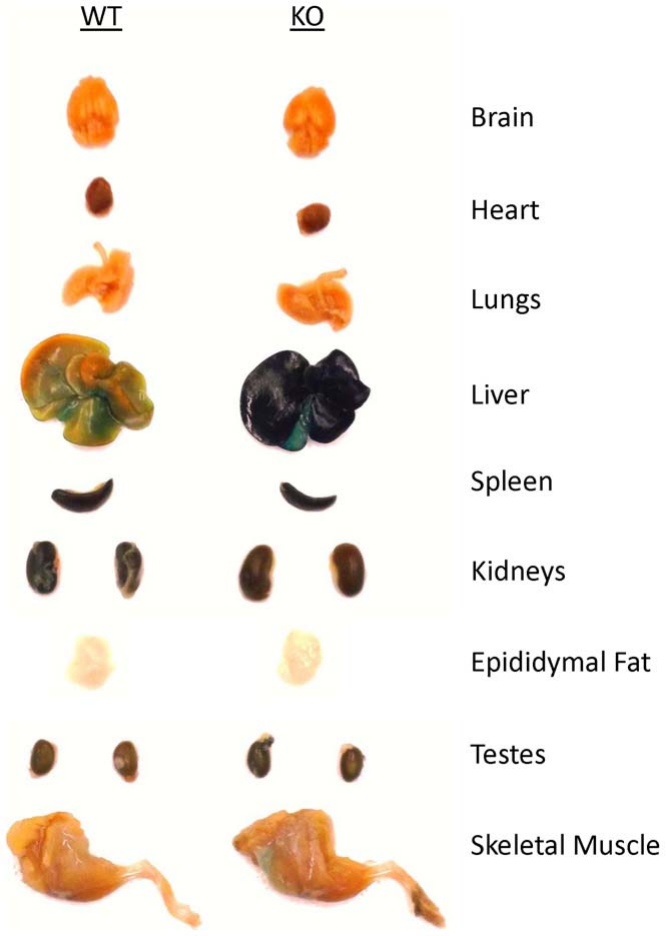
X-Gal staining of tissues from ApoF KO mice harboring a beta-galactosidase reporter gene. The targeted ApoF allele contains a beta-galactosidase reporter gene in place of ApoF. X-gal staining was performed on a wild type (WT) and an ApoF KO mouse (KO) to examine ApoF expression. The wild type mouse is included for an estimation of background staining for each tissue in the absence of beta-galactosidase.

Mouse apo F is highly homologous to human ApoF (61% identity at the amino acid level), and contains a consensus Furin cleavage site (RAKR↓S) where the proprotein region is cleaved to liberate the mature C-terminal fragment of the protein (Figure 2A). We found that mouse apo F utilizes a less common motif (NCS) for glycosylation at asparagine 206. It should be noted that this glycosylation site is in a different location than the N-linked site in the mature human apo F protein which lies at N267 (relative to the initiator Methionine) [14], [25]. We found that this glycosylation site at N206 in mouse apo F is nearly completely utilized when ectopically expressed in HEK293 cells (Figure 2B), as shown by the apparent 5 kDa drop in molecular weight when it is mutated to alanine. In addition to N-linked carbohydrate modification, human apo F is known to be extensively modified with *O*-linked sugars. Mature mouse apo F has a predicted molecular weight of 17.5 kDa for the mature protein, in contrast to the roughly 32 kDa band (excluding 5 kDa V5-His Tag) that we observe in conditioned media or mouse plasma. We treated plasma from mice overexpressing apoF using our liver-specific AAV-vector, to evaluate its size and glycosylation pattern (Figure 2C). Molecular weight was reduced in a stepwise fashion from 32 to 22 kDa when digested sequentially with PNGAseF, Sialidase A, and O-glycanase confirming that like human ApoF, mouse ApoF is also extensively modified with *O*-linked carbohydrate. Apo F is encoded by a small two exon gene located on mouse chromosome 10qD3, a region orthologous to human chromosome 12q13.3. The entire coding sequence and intron of the murine ApoF gene was replaced with a targeting cassette containing a B-galactosidase reporter gene (Figure 3A). The depicted sequence of Exon 1 and 2 represents remaining segments of the 5′ and 3′ untranslated regions respectively. Southern blotting was used to confirm the deletion and proper genomic location of the null allele. Digestion of the ApoF locus with BamHI yields a 3.9 kb fragment for the wild type allele, and a 2.4 kb fragment for the null allele when probed with a radiolabeled probe that hybridizes just downstream of the gene (Figure 3B). The defective ApoF allele was introduced into C57BL6-derived ES cells. The apo F mouse colony was generated and maintained on a pure C57BL6 background by breeding a male chimeric founder with C57BL6/J female mice and interbreeding the F1 progeny. Successful knockout of the apo F message was confirmed by realtime RT-PCR. As expected, apo F mRNA was reduced by 50% in the livers of the heterozygous mice, and was undetectable in the knockout mice (Figure 3C). Since the ApoF gene was replaced with a beta-galactosidase (B-Gal) reporter gene, we also performed X-gal staining to examine the expression pattern of B-Gal in tissues from adult mice (Figure 4). As expected from real time RT-PCR analysis for ApoF, impressive dark blue staining was evident in the livers of the ApoF KO mice. Some staining could be observed in the kidneys, testes and caudate epididymis after a prolonged incubation with the X-gal substrate, however these were equally blue in the WT control mice.

We next examined the plasma lipid phenotype of the ApoF deficient mice on a standard chow diet (Table 1). Female ApoF KO mice had total cholesterol, HDL cholesterol, non-HDL cholesterol, triglyceride, cholesteryl ester (CE) levels that were identical to wild type mice (Table 1). Female ApoF KO mice tended to have lower free cholesterol levels in the plasma, although this did not always reach statistical significance (KO: 32+/−9 vs. WT: 38+/−10 mg/dl, p>0.05). Female heterozygotes had plasma lipid values identical to wild type levels, with the exception of a very modest increase in CE content relative to that of the WT and KO mice (Het: 54+/−10 vs. WT: 45+/−9 mg/dl, p<0.05). Likewise, Male ApoF KO mice had total cholesterol, HDL cholesterol, non-HDL cholesterol, cholesteryl ester (CE), and free cholesterol (FC) values that were indistinguishable from wild type littermates. The male ApoF KO mice did have fasting triglyceride levels that were modestly but significantly lower than controls (KO: 33+/−11 vs WT: 43+/−18 mg/dl). The male ApoF heterozygous mice had marginally higher HDL cholesterol (Het: 70+/−7 vs. WT: 62+/−9 mg/dl), and cholesterol ester content (Het: 56+/−8 vs. WT: 50+/−7 mg/dl) relative to wild type mice. Body weight, liver weight, and liver somatic index were not significantly altered in the ApoF knockout mice (Table 1), and did not vary with aging. Interestingly, female ApoF KO mice consistently had higher liver cholesterol levels on a chow diet (KO: 4.9+/−0.8 mg/dl vs. WT 2.9+/−0.2 mg/dl, p<0.05). This was entirely attributable to an increase in cholesteryl ester content (KO 3.4+/−0.9 mg/dl vs. WT 1.2+/−0.3 mg/dl, p<0.05), while liver free cholesterol (FC) content was unchanged (Table 1). The male mice had a trend towards increased hepatic cholesterol content, however this did not reach statistical significance in any of our experiments (Table 1). Classical blood chemistry parameters including sodium, postassium, creatinine, blood urea nitrogen (BUN), aspartate aminotransferase (AST), and alanine aminotransferase (ALT) were not different by genotype, and fell within the expected reference intervals for mice (Table 2).

**Table 1:** Plasma lipid parameters in Apolipoprotein F knockout mice.

		Females			Males	
	WT	Het	KO	WT	Het	KO
Total Cholesterol (mg/dl)	83+/−9	87+/−17	81+/−13	93+/−10	96+/−9	93+/−12
HDL Cholesterol (mg/dl)	53+/−7	58+/−11	52+/−10	62+/−9	70+/−7*	64+/−8
Non-HDL Chol. (mg/dl)	30+/−5	29+/−6	30+/−8	31+/−8	26+/−8	29+/−8
Triglycerides (mg/dl)	28+/−10	22+/−9	26+/−10	43+/−18	31+/−12*	33+/−11*
Cholesteryl Ester (mg/dl)	45+/−9	54+/−10*	49+/−12	50+/−7	56+/−8*	53+/−9
Free Cholesterol (mg/dl)	38+/−10	33+/−10	32+/−9	43+/−7	40+/−9	39+/−9
Body Weight (g)	17.4+/−1.8	18.3+/−1.3	17.8+/−1.3	23.3+/−2.2	22.3+/−2.3	23.4+/−2.3
Liver Weight (g)	0.94+/−0.23	1.04+/−0.21	0.96+/−0.14	1.32+/−0.19	1.34+/−0.14	1.38+/−0.21
Liver: Body Weight %	5.3+/−0.8	5.7+/−1.3	5.4+/−0.6	5.7+/−0.5	6.0+/−0.4	5.9+/−0.8
Liver Cholesterol (mg/g)	2.9+/−0.2	2.7+/−0.3	4.9+/−0.8*	2.9+/−1.2	2.5+/−0.5	3.6+/−0.7
Liver Free Cholesterol (mg/g)	1.7+/−0.2	1.5+/−0.1	1.5+/−0.2	ND	ND	ND
Liver Cholesteryl Ester (mg/g)	1.2+/−0.3	1.2+/−0.4	3.4+/−0.9*	ND	ND	ND

Lipids were measured in fasting plasma from male Apolipoprotein F wild type (WT), heterozygous (Het), and knockout (KO) on a chow diet at 9–12 weeks of age. Data are reported as the mean +/− standard deviation for multiple mice of each genotype. Animal numbers are as follows: Plasma lipids n≥15 per group, Body and liver weights n≥5 per group, Liver cholesterol n≥4 per group. The asterisk (*) indicates p<0.05 relative to wild type by One way ANOVA, Tukey's posttest. ND indicates not determined.

**Table 2:** Blood Chemistry Parameters in ApoF KO mice.

	Females		Males		Reference Intervals for Mouse Blood
	WT *(n = 4)*	KO *(n = 5)*	WT *(n = 12)*	KO *(n = 6)*	
Sodium (mmol/L)	143+/−0.9	141+/−1.1	152+/−13	142+/−1.4	124–174
Potassium (mmol/L)	5.8+/−0.8	5.5+/−0.5	5.6+/−0.7	5.2+/−0.2	4.6–8
Creatinine (mg/dl)	0.2+/−0.0	0.2+/−0.0	0.2+/−0.0	0.2+/−0.0	0.2–0.8
BUN (mg/dl)	28+/−2.2	28+/−3.0	30+/−4.5	32+/−4.5	18–29
AST (U/L)	85+/−22	100+/−64	103+/−81	68+/−17	59–247
ALT (U/L)	46+/−6	60+/−11	51+/−12	45+/−7	28–132

Blood was collected in Lithium-Heparin tubes and used to measure classical chemistry parameters. Sodium, Potassium, Creatinine, Blood Urea Nitrogen (BUN), Aspartate Aminotransferase (AST), and Alanine Aminotransferase (ALT) were measured on a clinical chemistry analyzer. Data is reported as the mean +/− standard deviation. A two-tailed t-test was used to compare between genotypes by sex, and no statistically significant differences were found.

We made multiple attempts to evoke a lipoprotein phenotype in the ApoF KO mice using dietary manipulations. Female mice were fed a high fat (HF) diet containing 45% kCal from fat for 9 weeks. Plasma lipids were measured in the fasting state at baseline, and after 2, 4, and 9 weeks of HF feeding (Table 3). In this particular experiment, the female ApoF KO mice did exhibit significantly lower FC levels at baseline on the chow diet (KO: 23+/−2 vs. WT: 29+/−6 mg/dl, p<0.05). We observed robust increases in total cholesterol, HDL cholesterol, non HDL-C, CE, and FC after just two weeks of feeding which were maintained throughout the 9 week study for all genotypes (Time p<0.0001 by two way ANOVA). Surprisingly, the difference in FC seen in the ApoF KO mice was completely negated by the diet. At the two and four week time points, the ApoF Het and KO mice both had TG levels significantly lower than WT. There were no significant differences in weight gain, adipose tissue mass, or food consumption by genotype on the HF diet (data not shown). In further attempts to elicit a lipoprotein difference, we performed short term feeding to specifically test the effects of cholesterol loading or depletion (Table 4). Female mice were fed chow containing 0.2% cholesterol (w/w) or 0.01% ezetimibe (w/w) for 11 days. Neither dietary cholesterol challenge, nor cholesterol depletion with ezetimibe had any measurable impact plasma lipids; with the exception of a small but statistically significant difference in TG on the ezetimibe diet (KO: 30+/−4 vs WT: 38+/−6 mg/dl, p<0.05). Liver cholesterol levels trended higher in the ApoF KO relative to WT mice on both diets, but did not reach statistical significance in this experiment (Table 4).

**Table 3:** The effects of High fat feeding on plasma lipids in ApoF deficient mice.

	Genotype	Cholesterol (mg /dl)	HDL-C (mg/dl)	Non HDL-C (mg/dl)	Triglycerides (mg/dl)	Cholesteryl Ester (mg/dl)	Free Cholesterol (mg/dl)
	WT	79+/−6	53+/−5	26+/−2	25+/−6	50+/−5	29+/−6
Baseline	Het	79+/−7	54+/−6	25+/−2	18+/−6	54 +/−6	26+/−5
	KO	76+/−10	53+/−7	24+/−5	23+/−7	53+/−8	23+/−2*
	WT	126+/−8	95+/−6	31+/−5	25+/−27	89+/−10	37+/−8
2 weeks	Het	120+/−14	93+/−8	27+/−12	12+/−4*	81+/−13	40+/−7
	KO	121+/−11	92+/−10	29+/−3	11+/−1*	90+/−10	32+/−3
	WT	125+/−9	92+/−8	34+/−3	31+/−7	82+/−10	43+/−4
4 weeks	Het	122+/−10	90+/−7	33+/−5	16+/−4**	84+/−7	38+/−5
	KO	116+/−7	84+/−6	32+/−3	16+/−6**	79+/−6	37+/−2
	WT	131+/−8	98+/−7	33+/−5	21+/−8	82+/−8	48+/−3
9 weeks	Het	132+/−9	97+/−9	35+/−4	21+/−4	86+/−7	47+/−3
	KO	124+/−7	88+/−6	36+/−3	20+/−5	78+/−6	46+/−6

Female ApoF wild type (WT), heterozygous (Het), and knockout (KO) mice were bled for baseline lipid measurements and placed on a diet containing 45% kcal from fat for 9 weeks. Plasma was then collected after 2, 4, and 9 weeks on the diet. All mice were fasted 4 hours before bleeding at every time point. Values are reported as the mean +/− standard deviation for each group (WT n = 8, Het n = 10, KO n = 8).

**Two Way ANOVA Results:**

Cholesterol: Time p<0.0001.

HDL-C: Time p<0.0001.

Non HDL-C: Time p<0.0001.

Triglycerides: Time n.s.,Genotype p = 0.0025 (Bonferroni vs WT: * p<0.05, ** p<0.01).

CE: Interaction p = 0.0428, Time p<0.0001.

Free Chol: Time p<0.0001, Genotype p = 0.0003 (Bonferroni WT vs KO *p<0.05).

**Table 4:** The effect of cholesterol loading and depletion on plasma lipids in ApoF KO mice.

	0.01% Ezetimibe		0.2% Cholesterol	
	WT	KO	WT	KO
*“n”*	*6*	*6*	*4*	*5*
Total Cholesterol (mg/dl)	70+/−6	77+/−9	79+/−10	86+/−14
HDL Cholesterol (mg/dl)	45+/−4	49+/−4	45+/−4	49+/−5
Non HDL Cholesterol (mg/dl)	25+/−3	28+/−7	34+/−6	37+/−10
Triglycerides (mg/dl)	38+/−6	**30+/−4** *	23+/−4	24+/−5
Body Weight (g)	18.4+/−0.6	19.3+/−0.9	19.4+/−1.8	20.2+/−0.8
Liver Weight (g)	0.96+/−0.5	0.97+/−0.10	1.11+/−0.12	1.05+/−0.07
Liver : BW (%)	5.2+/−0.1	5.0+/−0.3	5.8+/−0.9	5.2+/−0.4
Liver Cholesterol (mg/g)	3.2+/−0.7	3.8+/−0.4	3.9+/−0.4	4.8+/−0.6

ApoF wild type (WT) and knockout (KO) mice (9–10 weeks of age) were fed a chow diet supplemented with either 0.01% ezetimibe or 0.2% cholesterol for 11 days. Values are reported as mean +/− standard deviation.

* P<0.05 relative to WT for each treatment group.

**Figure 5:**
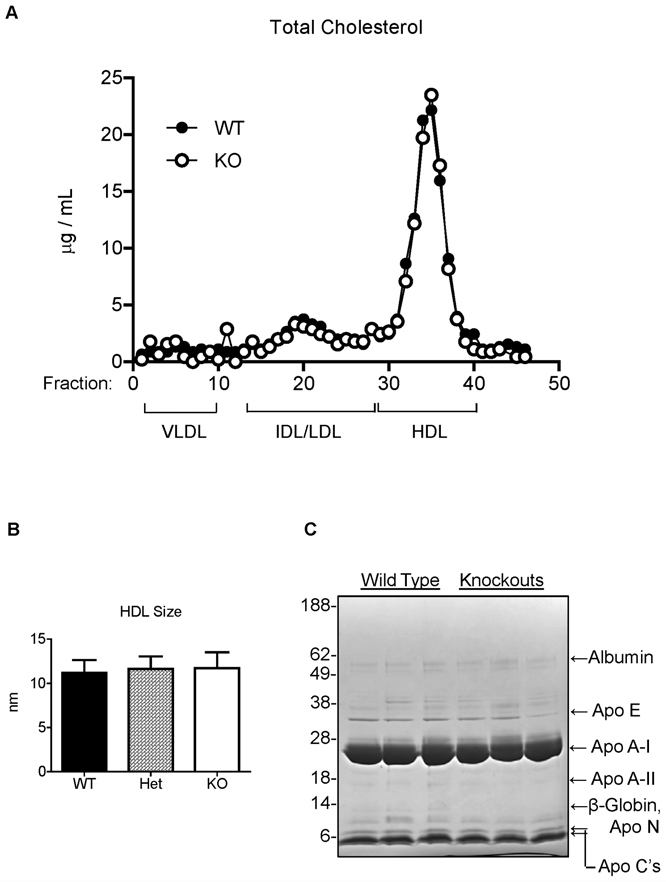
HDL cholesterol content, size, and apoprotein composition in ApoF KO mice. A. Gel filtration chromatography of 150 microliters of pooled plasma from wild type (WT), and ApoF KO (KO) mice. B. HDL size of plasma from wild type (WT), heterozygous (Het) and homozygous ApoF KO (KO) mice as determined by NMR analysis. C. Denaturing SDS-PAGE gel of proteins from HDL (1.063<d<1.21) centrifugally isolated from wild type and ApoF knockout mice. Ten micrograms of HDL protein was electrophoresed on 12% Bis-Tris gels and subjected to staining with Coomassie blue. The identity of albumin, apo E, apo A-I, apo A-II, and apo C's were determined by comparison to standards of human LDL, HDL and whole plasma. The 13 kDa band present in the wild type HDL but diminished in the KO was found to contain Apo N and B-globin as the primary protein components by LC-MS.

In human plasma, greater than 75% of the circulating apo F is found on HDL. We next examined the effect of apo F deficiency on HDL quantity, size and lipid composition. Gel filtration chromatography revealed a lipoprotein profile indistinguishable from that of the wild type mice (Figure 5A). The cholesterol content of the HDL peak was identical in height and area, and there was no shift that would suggest changes in particle size. Analysis of HDL particles by NMR confirmed that HDL size was unchanged in the knockout mice (Figure 5B). Next we isolated lipoproteins from these mice by KBr density gradient ultracentrifugation. There were no significant differences in the lipid or total protein content of HDL (1.063<d<1.21), nor were there differences in the composition of VLDL (d<1.006) or IDL/LDL (1.006<d<1.063) (Table 5). Lastly, we subjected the proteins from the isolated mouse HDL to SDS-PAGE to examine the apoprotein composition (Figure 5C). We observed no differences in the abundance of the major protein components of HDL. It should be noted that mouse ApoF should have a molecular weight of 32 kDa based on our overexpression studies. Endogenous apo F may exist below the limit of detection, or more likely is obscured by the far more abundant apo A-I band (29 kDa). One band that differed greatly between the groups at ∼13 kDa was excised from the gel for identification by LC-MS. This band was found to contain apolipoprotein N (apo N) and Beta-Globin as the main protein components.

**Table 5:** Lipoprotein Composition in ApoF KO mice.

Fraction	Genotype	Protein	Cholesteryl Ester	Free Cholesterol	Triglycerides	Phospholipids
VLDL	WT	37.4+/−4.8	1.6+/−0.3	1.6+/−0.1	55.2+/−5.3	4.3+/−1.3
	KO	44.3+/−6.2	1.4+/−0.1	1.3+/−0.4	49.3+/−6.7	3.7+/−1.2
IDL/LDL	WT	21.8+/−1.2	8.3+/−2.0	9.3+/−0.4	45.8+/−4.0	14.7+/−1.4
	KO	24.6+/−3.1	8.5+/−2.2	10.5+/−1.6	38.6+/−4.3	17.8+/−10
HDL	WT	48.6+/−0.9	22.7+/−1.1	5.6+/−0.2	0.62+/−0.06	22.4+/−0.4
	KO	47.7+/−1.1	20.8+/−2.4	5.9+/−0.8	0.72+/−0.21	24.9+/−2.7

Lipoproteins were isolated from one milliliter of pooled mouse plasma by KBr density gradient ultracentrifugation as follows: VLDL d<1.006, IDL/LDL 1.006<d<1.063, HDL 1.063<d<1.21 g/mL. Concentrations for each lipid are given as the percentage of total mass in the fraction. Values are reported as the mean +/− standard deviation of three separate isolations (n = 3), with each value representing a separate and distinct pool of at least four mice per genotype.

**Figure 6:**
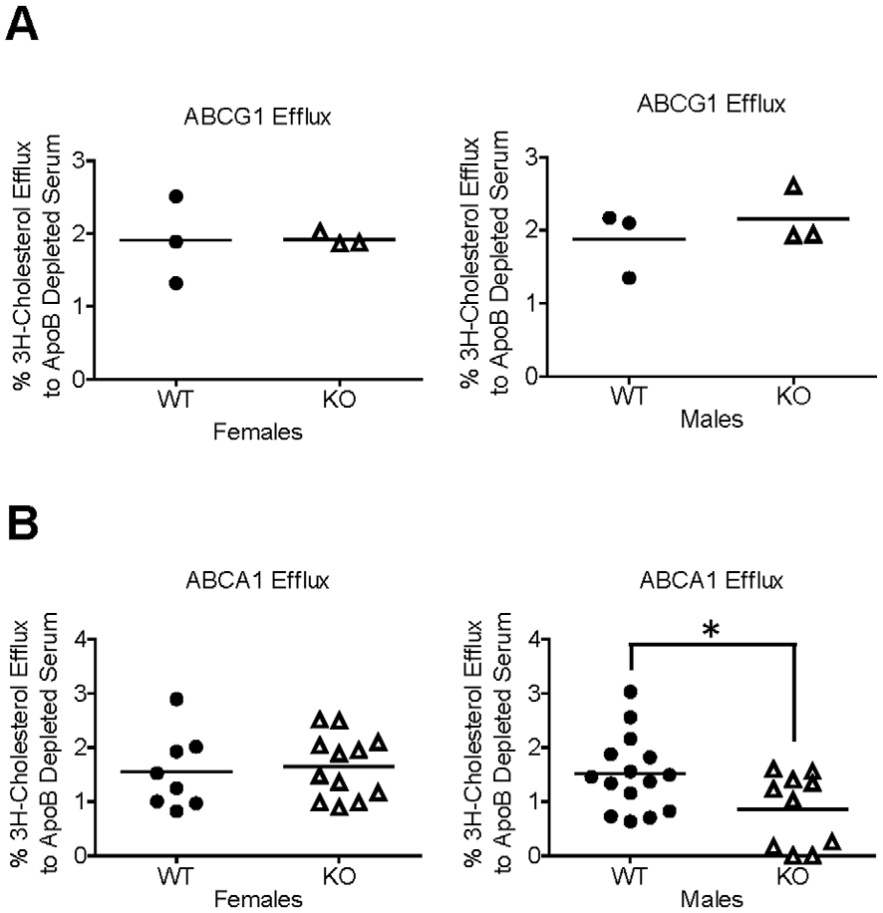
Cholesterol efflux capacity of HDL fractions from ApoF KO mice. Serum was collected from wild type and ApoF KO mice for the measurement of cholesterol efflux capacity.. Apo B lipoproteins were precipitated with polyethylene glycol, and the resulting supernatant (containing HDL and other soluble serum proteins) was tested for its ability to promote cholesterol efflux as described in the methods section. *A.* ABCG1-mediated cholesterol efflux to apo B-depleted serum from wild type (WT) and ApoF knockout (KO) mice. *B.* ABCA1-mediated cholesterol efflux.

We previously reported that HDL from mice overexpressing ApoF had an increased ability to accept macrophage derived cholesterol on a per-particle basis. To test the relevance of these findings at physiological levels of ApoF expression, we performed efflux studies on apo B-depleted serum obtained from the ApoF knockout mice. To examine ABCG1-mediated efflux, we used BHK cells stably transfected with ABCG1 driven by a mifepristone-inducible promoter [26]. In this cell system, ABCG1-mediated efflux is assessed as the difference between cells treated with and without mifepristone. ABCG1-mediated efflux was not significantly different between wild type and ApoF KO mice in either sex (Figure 6A). To assess ABCA1-mediated efflux, we used J774 macrophages treated with cyclic AMP to upregulate ABCA1 expression. ABCA1-mediated efflux was calculated as the difference in efflux between cells treated with cAMP, and wells treated without cAMP for each sample. ABCA1-mediated efflux was not significantly altered in the female ApoF KO mice. Surprisingly, we did observe a modest but statistically significant reduction in ABCA1-mediated efflux in samples from the male ApoF KO mice relative to control animals (↓0.66%, p = 0.027).

## Discussion

We generated mice with a targeted deletion of the ApoF gene in order to investigate its effects on HDL cholesterol metabolism. ApoF KO mice were viable and fertile, and had no gross changes in HDL cholesterol levels, size, or lipid composition on a chow diet. This is perhaps a bit surprising since we previously found that overexpression of apoF reduces HDL-C in mice by accelerating clearance from the circulation [14]. The lack of an HDL phenotype in the knockout mice suggests that either 1) apo F is not a major player in HDL metabolism *in vivo* at physiological levels of expression, or 2) less likely, compensatory changes or redundancy mask the impact of apo F deficiency. We have also attempted to evoke an HDL phenotype using a variety of dietary manipulations including high fat diet, high cholesterol diet, and ezetimibe feeding. In all cases examined, HDL-C and other plasma lipid levels did not substantially vary by genotype. It should be noted that apo F is known to be repressed by high fat feeding in mice [27], as well as hamsters [17]. While high fat feeding is a straight forward way to increase HDL in mice, transcriptional repression of apo F by high fat could be simplistically expected to diminish any lipid differences between WT and KO mice. Likewise, dietary cholesterol loading and sterol depletion with ezetimibe both failed to reveal lipid differences by genotype. Since about 25% of the circulating apo F is also found on LDL in human plasma, additional studies may be warranted to examine the effects of apo F on apo B lipoprotein metabolism in other mouse models such as LDL receptor knockout or ApoE knockout mice.

Mouse apo F shares considerable homology (61% AA identity) with human apo F, and is processed and secreted in a similar manner. In agreement with findings in other species [5], [17], mouse apoF was expressed at far greater levels in liver than in any of the other whole tissues examined. It is possible that local expression of ApoF may play an important role in steroidogenic or exocrine tissues, and this may warrant further investigation in specific cell populations. In terms of the plasma pool of ApoF, the data suggests that the liver is by far the most important contributor to ApoF mass present on circulating lipoproteins. We found that mature mouse apo F exists in plasma as an approximately 32 kDa protein, and is modified with both N- and O-linked sugars. Mouse apo F has a single N-linked glycosylation site (N206) at a different location in the mature protein than human apoF (N267). Although the proteins are fairly well conserved at the amino acid level, differences in glycosylation patterns may hint at a divergence in function.

In humans, apo F exists in plasma at concentrations in the range of 80 µg/ml (similar to that for apo E), of which ∼60 µg/mL should reside on HDL. Assuming a plasma protein concentration of 1.34 g/L apoA-I for men [28], and an average of roughly 2.5 molecules of apoA-I per HDL particle [29], we estimate that a theoretical maximum of 1 in 9 HDL particles could potentially contain ApoF. While not exceedingly abundant, this is in the range where apo F could realistically be expected to alter HDL levels- either by affecting receptor mediated uptake, or by influencing lipid transfer in or out of the particle. We were not able to detect endogenous mouse apo F, and therefore cannot estimate the normal abundance of this protein in mouse plasma. Our inability to detect endogenous mouse ApoF in plasma may be due to limitations of the antibody, which was raised in rabbits against a bacterially expressed mouse apo F proprotein. Since sugar accounts for greater than half the apparent molecular weight of apo F, it is plausible that the epitopes of plasma apo F recognized by the antibody may be covered up or otherwise sterically obstructed by carbohydrate.

We previously found that plasma from mice overexpressing apo F was more efficient at promoting macrophage cholesterol efflux on a per-particle basis [14]. ABCG1-mediated efflux was not significantly different in ApoF KO mice of either sex. Interestingly, ABCA1-mediated efflux was modestly but significantly lower in the male ApoF KO mice relative to WT controls. This relatively small difference, albeit sex-specific to males, is consistent with our previous results obtained in the setting of ApoF overexpression. It is worth noting that the absolute amount of ABCA1-mediated efflux from J774 macrophages to mouse plasma was in the range of 0 to 2%, in contrast to total efflux values of 10–12% in the presence of cAMP. This was a consistent result and may be due to a relatively low concentration of pre-beta HDL in mouse plasma compared to human plasma. It is not clear how loss of ApoF, a protein normally found on larger HDL (but oddly enough of HDL3 density), would affect ABCA1-mediated efflux.

The reduced ABCA1-mediated efflux in the male ApoF KO mice may suggest a potential protective role for ApoF in atherosclerotic disease. However, without information on the absolute abundance of mouse apoF in plasma, it is difficult to extrapolate these data to the human situation. At the population level, variation in the CETP gene is the single greatest genetic determinant of HDL cholesterol levels in the humans [30]. The role of ApoF in lipid metabolism and atherosclerotic disease may be very different in humans, where apo F is believed to be an endogenous inhibitor of CETP activity. It was recently shown that apo F levels in HDL were significantly reduced in a small set of subjects with CAD [31]. In this same report, apo F protein levels were increased after one year of combined statin and niacin treatment, suggesting apo F is associated with a favorable proteomic signature for HDL. Variation within the human ApoF gene is rare in most populations, but a systematic analysis of ApoF genetic variants, proteins levels, and CAD status in a large cohort is clearly warranted to inform about the biology of this cryptic protein in humans.

Two previous studies have implicated variation at the ApoF locus in mouse physiology. A genetic screen between SM and NZB inbred mice identified a region near the ApoF gene at *D10Mit271* as a quantitative trait locus (QTL) for PLTP activity [27]. Although coding differences between the strains were observed for ApoF, its candidacy as the gene underlying this QTL is questionable considering the proximity of this linkage peak to the Farnesoid X Receptor (FXR) gene, whose expression varies 10-fold between the strains. FXR is a nuclear receptor known to directly activate transcription of the PLTP gene [32]. PLTP deficiency is known to have major effects on HDL size and quantity in mice [33]. If apo F were a *bona fide* inhibitor of PLTP transfer in mice, we would have expected to observe at least a subtle effect on HDL size or lipid composition.

Another study examined the genetic determinants of leukemia virus inactivating factor (LVIF) activity in mice [34]. LVIF is a believed to be a lipoprotein associated protein with the ability to inactivate murine leukemia viruses- either by itself or with the particle on which it resides [35], [36], [37]. An intercross between strains with and without serum LVIF activity identified a strong single linkage peak at *D10Mit35*, near the ApoF gene. However, apo F expression did not vary between the strains and sequencing revealed no differences in the coding regions of apo F. The authors suggested that LVIF activity is likely under the control of a distinct, but closely linked gene. It is interesting to note that Apolipoprotein N (Apo N) resides only 12 kb upstream of ApoF in the mouse. Apo N has been described as an HDL-associated protein that bears sequence homology to apo F in the proprotein region, although the mature form of apo N has no similarity to mature apo F. Interestingly, apo N is a nonfunctional pseudogene in humans due to a frameshift mutation that gives rise to a stop codon before the mature protein. It is possible that apo N may be the true LVIF gene in mice. It is also tempting to speculate that functional redundancy may exist between apo F and apo N, and that they may reside on HDL to perform similar functions. Further work is needed to explore potential roles of these genes in inflammation and innate immunity. We conclude that mouse apo F is not a major player in HDL metabolism in mice, and it likely resides on the HDL particle for a reason distinct from lipid transport. The apo F deficient mice will be an invaluable tool in deciphering the true function of apoF-containing HDL, and offer important clues about the role of apo F in cardiovascular disease.

## Materials and Methods

### Ethics Statement

All animal procedures were performed according to the regulations of, and with the prior approval of, the University of Pennsylvania Animal Care and Use Committee (IACUC)- protocol number 706003.

### Generation of Apolipoprotein F Deficient Mice

We obtained embryonic stem cells harboring a null ApoF allele from the knockout mouse project (www.komp.org). The ZEN-Ub1 cassette, which contains a Beta Galactosidase Reporter gene followed by a Neomycin resistance gene driven by the phosphoglycerate kinase (PGK) was inserted into the ApoF locus, such that the entire coding sequence and single intron of the gene were replaced. This was done in VGB6 ES cells, which are derived from C57BL/6NTac mice. We obtained three clones termed 10417A-D6, 10417B-H8, and 10417A-H2- which were determined to be at least 79%, 68% and 45% euploid respectively. The ES cells were expanded in growth media containing 15% ES grade serum (Hyclone), 2 mM L-glutamine, 1 mM Non-essential amino acids, 1 mM sodium pyruvate, 8 µl/ml β-mercaptoethanol, 1×10^7^ U/L Leukemia Inhbitory Factor (LIF) from ESGRO, and 1× Penicillin/Streptomycin cocktail in DMEM (Gibco). ES cells were plated on top of feeder layers of primary mouse embryonic fibroblasts (Millipore, PMEF-N), in 6-well surface modified polystyrene plates (Beckman 353846), and were split at least once every three days. Following confirmation of genotype by Southern blotting, the ES cells were delivered to the Penn Transgenic and Chimeric Mouse Facility for Microinjection. Clone 10417A-D6 was injected into 41 Balb/c blastocyts which were implanted into four pseudopregnant recipients. Three male chimeras were obtained- 90%, 85%, and 70% by coat color, and mated with C57BL6/J female mice. One chimeric mouse (90% chimerism) successfully transmitted the null ApoF allele in the germline and was used to found a colony by breeding to multiple C57BL6/J females. ApoF knockout mice were viable and obtained in the expected 1∶3 Mendialian ratio from breedings of heterozygous mice. No breeding or fertility problems were observed in the homozygous null mice. All wild type C57BL6 control mice were bred and maintained in house, and matched for sex and age in all experiments. No differences in lipids were observed between WT or ApoF KO mice obtained by Het to Het breeding versus those obtained from homozygote to homozygote breeding.

### Animals

Mice were housed in an AAALAC accredited animal facility in polycarbonate cageswith filter tops. Mice were maintained on a light cycle from 6 am to 6 pm, and had free access to food and water. Mice were maintained on a chow diet (Purina LabDiet 5010) unless otherwise indicated. Mice were also fed a high fat diet containing 45% kcal from fat (D12451i, Research Diets) for 9 weeks. Other mice were fed or Purina LabDiet 5010 supplemented with either 0.2% cholesterol (Research Diets C16014) or 0.01% ezetimibe (Research Diets C16012i) for 11 days as indicated in the text. Mice were fasted for four hours prior to sacrifice and bleeding for lipid analysis. Animals were sacrificed at 10–12 weeks of age, with the exception of those animals challenged with a high fat diet, ezetimibe diet, or cholesterol-enriched diet. In these cases, feeding began at 10–12 weeks of age, and the mice were sacrificed immediately after the indicated feeding regimen. Blood was collected in heparinized Natelson collection tubes by retro-orbital bleeding. Plasma was isolated by centrifugation at 16,000 g for 7 minutes at 4°C in a bench top microcentrifuge.

### Southern Blotting

Genomic DNA was isolated from confluent wells of ES cells by lysis in a buffer containing 100 mM EDTA, 60 mM Tris pH 7.5, 0.1% SDS, and 200 µg/mL Proteinase K. DNA was extracted by adding an equal volume of phenol pH 7.0 (Ameresco), and an equal volume of chloroform∶isoamyl alcohol (24∶1) in a 50 mL polypropylene conical. The same lysis buffer was used to isolate genomic DNA from mouse livers following ultracentrifugal isolation of nuclei as previously described [38]. Samples were gently rocked for 10 minutes and then centrifuged at 2000 rpm in a bench top centrifuge to separate the phases. The viscous aqueous upper phase was transferred using a cut off 1 mL pipet tip to a new 50 mL conical tube. The DNA was then re-extracted with 2 volumes of chloroform isoamyl alcohol (24∶1). One tenth volume of 3M sodium Acetate (pH 7.0) was added to the DNA and thoroughly mixed in by swirling. The DNA was layered with 2.5 volumes of ice cold ethanol, and spooled by rapid swirling with a hooked Pasteur pipet at the water/ethanol boundary. The spooled DNA was removed from the Pasteur pipet by appling 1–2 mL of TE buffer (10 mM Tris pH 7.5, 0.1 mM EDTA) dropwise with a 1 mL pipetman. DNA was dissolved by gentle rocking overnight at 4°C. Aliquots of the DNA were digested overnight at 37°C with 10 Units/µl of *BamHI* (New England Biolabs) in the buffer recommended by the manufacturer. Fifty micrograms of DNA were electrophoresed on a large 0.9% agarose gel in Tris Acetate EDTA (TAE) running buffer. The gel was stained with Ethidium bromide, photographed, and transferred by upward capillary transfer to a Nylon membrane. A short DNA probe corresponding to a 314 bp sequence downstream of the ApoF gene was obtained by PCR using the following primers: Forward 5′-GGT CCT TGA ACC GCT TGG AGA ATT G-3′, Reverse 5′-GGC TTA TGA CAA GGA TCA TAA TGG GTA CAT AG-3′. Fifty nanograms of probe DNA was labeled with 50 µCi of ^32^P-alpha-dCTP using the High Prime Labeling Kit (Roche). The membrane was hybridized with the denatured DNA probe in ExpressHyb Solution (Clontech) containing 0.2 mg/mL sonicated salmon sperm DNA (Stratagene) at 50°C for 2 hours. The membrane was then washed twice for 10 minutes each in 2× Sodium Chloride/Sodium Citrate (SSC) with 0.5% SDS, followed by two washes at 50°C in 0.1× SSC with 0.1% SDS. Membranes were then allowed to expose autoradiography film (Kodak X-OMAT LS) with a BioMax intensifying screen at −80°C for 1–3 days.

### RNA Isolation and Realtime RT-PCR

Approximately 20 milligrams of liver was placed into 2.0 mL cryotubes (Sarstedt) and rapidly flash frozen in liquid nitrogen. A steel bead and 1 mL of Trizol reagent (Invitrogen) were added to each cryotube. The livers were homogenized using a TissueLyzer bead mill homogenizer (QIAGEN) for 5 minutes at 25 Hz. Total RNA was isolated according to the standard Trizol procedure (Invitrogen). RNA concentrations and purity were determined using a UV NanoSpec device. One microgram of total RNA was reverse transcribed using random hexameric primers with the Superscript III RT Kit from Invitrogen according to the manufacturer's instructions. Predesigned TaqMan primer/probe sets from Applied Biosystems were used to detect mouse ApoF (Mm00506066_g1) and Beta Actin (4352341E). Reactions were set up containing 12.5 µl of 2× Taqman reaction mix, 0.5 µl of primer/probe, and 5 µL cDNA (diluted 1∶20) in a final volume of 25 µL. PCR conditions were 95°C for 5 minutes, then 95°C for 1 minute and 60°C for 30 seconds, cycled 40 times. The relative quantity of ApoF mRNA was calculated using the delta delta CT method with B-Actin as the housekeeping gene.

### X-Gal Staining of Tissues

Whole tissues were harvested from mice after perfusion with PBS. The individual tissues were separately placed in 20 ml glass vials and fixed in 10 volumes of 2% paraformaldehyde (Electron Microscopy Services) for one hour with rocking at 4°C. The tissues were then washed 6 times with 10 volumes of PBS for 20 minutes each. Tissues were then incubated with the X-gal substrate overnight at 37°C in a buffer containing PBS with 0.1% deoxycholic acid, 2 mM MgCl_2_, 5 mM Potassium Hexacyanoferrate (iii) (Sigma 244023), 5 mM Potassium Hexacyanoferrate (ii) (Sigma P9387), 0.1% NP40, and 1 mg/ml X-gal.

### Cell Culture

Adherent HEK293 cells (obtained from the American Type Culture Collection; www.atcc.org) were grown in DMEM with 10% FBS, and 1% Pen/Strep as previously described [39]. Confluent wells of HEK293 cells in 6 well plates were transiently transfected Lipofectamine 2000 according to the manufacturer's instructions. The day after the transfection, media was switched to OPTI-MEM low serum media (Invitrogen). Media was collected 48 hours later and frozen. Cells were washed 2× with PBS, and lysed in 1 mL of 1× LDS buffer (Invitrogen). Cell lysates were freeze-thawed and vortexed to clear debris, and then boiled and subjected to Western Blotting.

### Enzymatic Deglycosylation

Plasma was obtained from mice overexpressing mouse ApoF two weeks after the administration of 5×10^11^ genome copies (GC) of our previously described AAV8-TBG-mApoF vector [14]. Enzymatic deglycosylation of plasma proteins was accomplished using the PROZyme kit from Glyko (catalog number GK80115). Briefly, 7 µL of plasma was diluted with 10 µL of 5× incubation buffer and 2.5 µL denaturation solution to a final volume of 32.5 µL with water. The samples were denatured at 100°C for 5 minutes in boiling water, and allowed to cool. Detergent solution (2.5 µL) was added to each tube and mixed. Next, PNGAse F (1 µL), Sialidase A (1 µL), and O-glycanase (1 µL) were added to each tube in the combinations shown in Figure 1, with the remaining volume filled with water. The denatured samples were digested for 3 hours in a 37°C water bath. The reactions were terminated with the addition of 50 µL of 2× Western Sample Buffer (6 M Urea, 3% SDS v/v, 0.2 M sucrose, 5% β-mercaptoethanol, 60 mM Tris pH 6.8, and 0.005% bromophenol blue) and immediately frozen until Western blotting was performed.

### SDS-PAGE and Western Blotting

Cell lysates prepared in 1× LDS sample buffer were aliquotted and then received 1/10^th^ volume of 10× reducing agent (Invitrogen). Conditioned media collected from cells was combined with 4× LDS sample buffer and 10× reducing agent to volume. Dialyzed HDL (10 µg protein/lane) was diluted with 4× LDS sample buffer and run without reducing agent. Samples were denatured by boiling in water for 5 minutes and loaded (fifteen microliters per well) on NuPAGE precast Bis-Tris gels, and electrophoresed in 1× MES running buffer (Invitrogen). Proteins were transferred to nitrocellulose membranes and blocked for one hour in blocking milk (5% Nonfat dry milk-Carnation, 0.05% Tween 20, PBS). Antibodies were added for 2 hours to overnight in blocking milk. The primary antibodies were removed by three 10 minute washes in PBS with 0.05% Tween 20 (PBS-T). Donkey anti-rabbit Horseradish Peroxidase Conjugated IgG (GE Healthcare) was added for two hours at a 1∶4000 dilution in PBS-T. The secondary antibody was removed with 3×10 minute washes with PBS-T. Membranes were developed by chemiluminescene using the ECL reagent from GE, and allowed to expose film. The rabbit anti-mouse apo F antibody was a kind gift from Dr. Kaijin Wu and Dr. Sarah Hamm-Alvarez (University of Southern California). This antibody was raised against the entire proprotein region of mouse ApoF that was expressed in E. Coli and affinity purified [40]. Rabbit anti-mApoF was used at a 1∶4,000 dilution. HDL gels were stainined with 0.1% Coomassie brilliant blue in methanol-acetic acid water (3∶1∶6) as previously described [41].

### Lipid and Lipoprotein Analyses

Plasma lipids were analyzed on a Roche Cobas-Mira autoanalyzer using commercially available reagents from Sigma. For gel filtration chromatography, one hundred and fifty microliters of pooled mouse plasma was diluted in 150 mM NaCl w/ 1 mM EDTA and separated on over two tandem Superose 6 gel filtration columns (GE Healthcare) using an AKTA FPLC system as previously described [42]. One hundred microliters of each 500 µL fraction was assayed for cholesterol content using the WAKO Cholesterol E kit. Nuclear magnetic resonance (NMR) analysis of plasma was carried out by LipoScience.

### Liver Cholesterol Measurements

Liver pieces (250 mg) were homogenized in 750 µL of PBS using a Tissuelyzer bead mill homogenizer (QIAGEN). The liver lysates were then diluted 1∶5 with PBS by combining 200 µL of homogenate with 800 µL PBS. Twenty microliters of the diluted lysate was pipeted into each well of a 96 well microtiter plate (Polysorp, NUNC). Lipids were solubilized at 37°C for 5 minutes with the addition of 20 µL of 0.25% deoxycholic acid solution. Human serum standards of known cholesterol concentration were prepared and solubilized in parallel. Infinity Cholesterol Reagent (200 µL) was added to each well containing standard or liver lysate, and incubated for 15 minutes at 37°C. The absorbance of each well was read at 500 nm on a microplate reader. The concentration of cholesterol (in units of mg cholesterol/g liver) was calculated by extrapolating from the standard curve [43]. Liver free cholesterol content was determined according to the method of Carr and Rudel [44], using the Wako Free Cholesterol E reagent for quantitation. Liver cholesteryl ester content was determined by subtracting the free cholesterol from total cholesterol.

### holesterol Efflux Assays

Serum or plasma was collected from wild type and ApoF knockout mice. ApoB lipoproteins were precipitated as previously described [45]. Briefly, serum was mixed with 0.4 volumes of PEG solution containing 20% PEG 8000 MW (Sigma P-2139) and 200 mM Glycine pH 7.4. The precipitation reactions were incubated at room temperature for 20 minutes, and then centrifuged at 16,000 g for 30 minutes at 4°C. The resulting supernatant, containing HDL and other serum proteins, was set aside for efflux assays. Cholesterol efflux assays were performed as previously described [46], [47]. J774 cells were used to assess ABCA1 mediated efflux. J774 cells (obtained from the American Type Culture Collection; www.atcc.org) were grown in RPMI media containing 20% FBS at plated at a density of 70,000 cells per well in 24-well plates as previously described [46]. Cells were labeled for 24 hours with 2 µCi/ml [1,2-^3^H] cholesterol (PerkinElmer) in 5% FBS-RPMI. Following labeling, J774 cells were treated +/−0.3 mM c-AMP (cpt-AMP, Sigma) in 0.2% BSA for 16–18 h to upregulate ABCA1. Efflux to 2.8% PEG supernatants (ApoB depleted sera) in RPMI with 0.2% BSA was performed for four hours. All reactions were performed in triplicate, and the values were averaged for each sample. ABCA1-mediated efflux was calculated as the difference between cells treated with cAMP and those treated without cAMP. ABCG1 mediated efflux was measured using BHK cells stably transfected with ABCG1 under the control of a mifepristone inducible promoter [26] (gift from Dr. Jack Oram, University of Washington School of Medicine). BHK cells were plated in 24 well plates at a density of 125,000 cells per well. Following labeling with 1 µCi/ml [1,2-^3^H] cholesterol, the cells were treated for 16 hours with 10 nM mifepristone to upregulate ABCG1 expression. Efflux was performed over 4 hours to 2.8% PEG supernatant (equivalent to 2% serum) in RPMI with 0.2% BSA. ABCG1 mediated efflux was calculated as the difference in efflux between cells with mifepristone and those without.

### Statistical Analyses

Comparisons between groups were made using a two-tailed homeoscedastic student's t-test. Comparisons involving three groups or more were made by ANOVA, followed by Tukey's post test to compare between groups where differences existed. P values less than 0.05 were considered statistically significant, and are noted with an asterisk (*) in the figures. For comparisons involving multiple groups and time points, two way ANOVA was performed, followed by Bonferroni's posttest to compare to the wild type where differences existed.

## Supporting Information

Figure S1 **Real time RT-PCR standard curves.** Liver cDNA was pooled and serially diluted 1∶4 to determine the log-linear range of the assay. The diluted cDNA was used as a template to measure ApoF or B-Actin expression with Taqman primers and probes as described in the methods section. The threshold cycle (Ct) is shown on the y-axis, and the relative quantity of cDNA template is shown on the x-axis. A linear regression was performed to determine the log-linear range of the assay. *A.* ApoF Taqman primer and probe *B.* B-Actin Taqman primer and probe.
(TIFF)

Table S1 **Threshold Cycles for ApoF and Beta Actin in male wild type mice.** One microgram of RNA was reverse transcribed to cDNA in a final volume of 20 µL. The resulting cDNA was diluted 1∶25 in the final reaction volume. Relative quantities were calculated using the delta delta Ct method, and are given to relative liver which is normalized to an arbitrary value of 1,000,000. Relative quantities are reported as the mean +/− standard deviation for each tissue.
(PDF)

Table S2 **Threshold Cycles for ApoF and Beta Actin in female wild type mice.** One microgram of RNA was reverse transcribed to cDNA in a final volume of 20 µL. The resulting cDNA was diluted 1∶156 in the final reaction volume. Relative quantities were calculated using the delta delta Ct method, and are given to relative liver which is normalized to an arbitrary value of 1,000,000. Relative quantities are reported as the mean +/− standard deviation for each tissue.
(PDF)
