# Supplementary figures and images for: The Effects of Apolipoprotein F Deficiency on High Density Lipoprotein Cholesterol Metabolism in Mice
Source: PLoS One. 2012 Feb 20;7(2):e31616. doi: 10.1371/journal.pone.0031616 (PMC3282742; doi:10.1371/journal.pone.0031616)

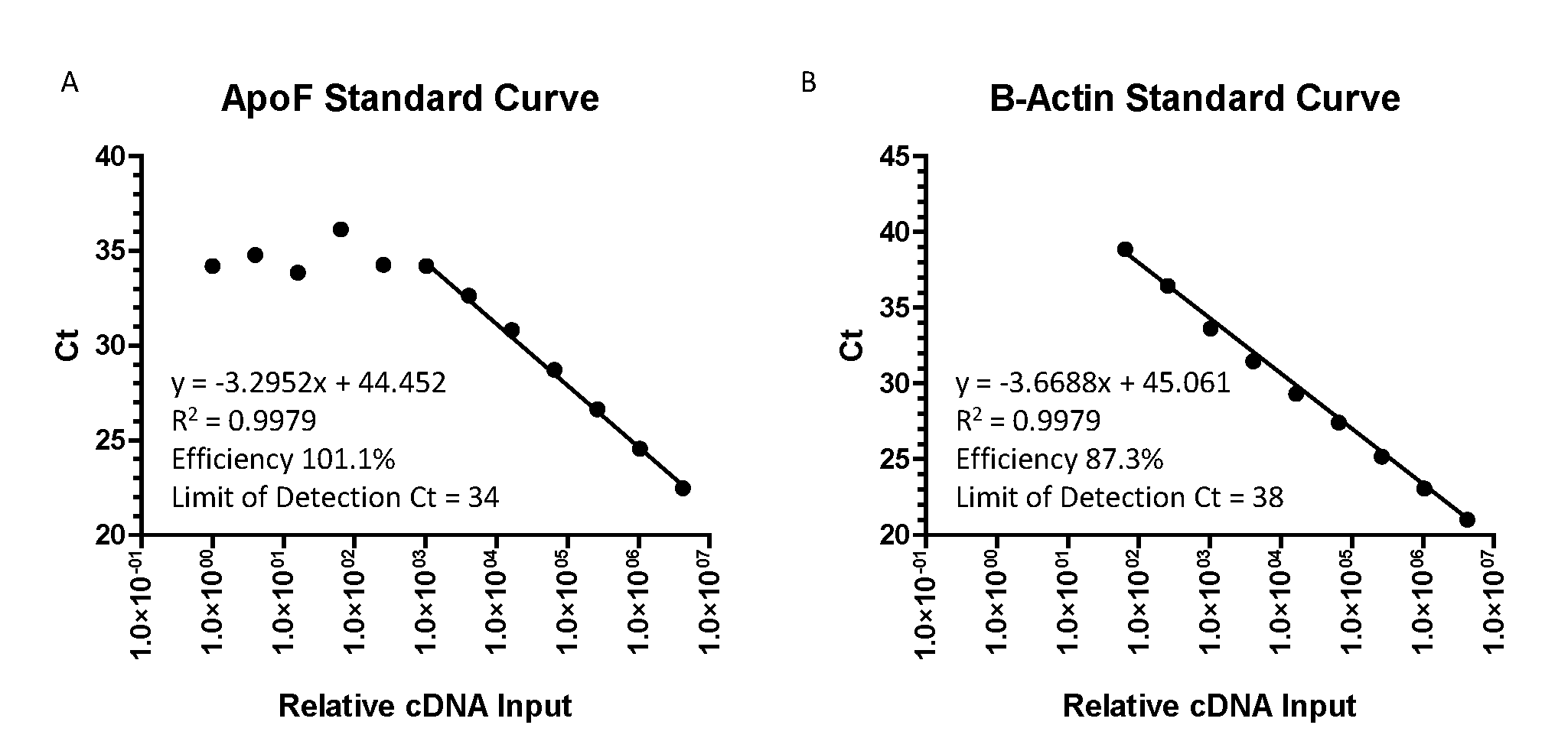

Supplement: Figure S1 — Real time RT-PCR standard curves. Liver cDNA was pooled and serially diluted 1∶4 to determine the log-linear range of the assay. The diluted cDNA was used as a template to measure ApoF or B-Actin expression with Taqman primers and probes as described in the methods section. The threshold cycle (Ct) is shown on the y-axis, and the relative quantity of cDNA template is shown on the x-axis. A linear regression was performed to determine the log-linear range of the assay. A. ApoF Taqman primer and probe B. B-Actin Taqman primer and probe. (TIFF) [file pone.0031616.s001.tiff]
