# Supplementary material for: The Effects of Apolipoprotein F Deficiency on High Density Lipoprotein Cholesterol Metabolism in Mice
Source: PLoS One. 2012 Feb 20;7(2):e31616. doi: 10.1371/journal.pone.0031616 (PMC3282742; doi:10.1371/journal.pone.0031616)
Supplement: Table S1 — Threshold Cycles for ApoF and Beta Actin in male wild type mice. One microgram of RNA was reverse transcribed to cDNA in a final volume of 20 µL. The resulting cDNA was diluted 1∶25 in the final reaction volume. Relative quantities were calculated using the delta delta Ct method, and are given to relative liver which is normalized to an arbitrary value of 1,000,000. Relative quantities are reported as the mean +/− standard deviation for each tissue. (PDF) [file pone.0031616.s002.pdf]

Supplementary Table S1- Threshold Cycles for ApoF and Beta Actin in male wild type mice. One microgram of RNA was reverse transcribed to cDNA in a final volume of 20 µL. The resulting cDNA was diluted 1:25 in the final reaction volume. Relative quantities were calculated using the delta delta Ct method, and are given to relative liver which is normalized to an arbitrary value of 1,000,000. Relative quantities are reported as the mean +/- standard deviation for each tissue.

TISSUE	ApoF Ct	B-Actin Ct	Relative Qty
Brain	33.3	21.2	215 +/- 157
Heart	34.6	23.6	1404 +/- 2178
Lung	34.3	22.0	183 +/- 131
**Liver**	**21.9**	**22.2**	**1000000 +/- 294856**
Spleen	31.7	21.2	573 +/- 249
Kidney	34.1	22.2	316 +/- 255
Duodenum	34.4	20.6	68 +/- 55
Jejunum	35.3	19.4	15 +/- 11
Ileum	33.9	18.7	43 +/- 59
Inguinal Fat	37.4	21.5	26 +/- 29
Brown Fat	37.5	23.7	125 +/- 175
Stomach	32.5	22.2	714 +/- 534
Testes	30.9	21.8	1415 +/- 265
