# Supplementary material for: The Effects of Apolipoprotein F Deficiency on High Density Lipoprotein Cholesterol Metabolism in Mice
Source: PLoS One. 2012 Feb 20;7(2):e31616. doi: 10.1371/journal.pone.0031616 (PMC3282742; doi:10.1371/journal.pone.0031616)
Supplement: Table S2 — Threshold Cycles for ApoF and Beta Actin in female wild type mice. One microgram of RNA was reverse transcribed to cDNA in a final volume of 20 µL. The resulting cDNA was diluted 1∶156 in the final reaction volume. Relative quantities were calculated using the delta delta Ct method, and are given to relative liver which is normalized to an arbitrary value of 1,000,000. Relative quantities are reported as the mean +/− standard deviation for each tissue. (PDF) [file pone.0031616.s003.pdf]

Supplementary Table S2- Threshold Cycles for ApoF and Beta Actin in female wild type mice. One microgram of RNA was reverse transcribed to cDNA in a final volume of 20 µL. The resulting cDNA was diluted 1:156 in the final reaction volume. Relative quantities were calculated using the delta delta Ct method, and are given to relative liver which is normalized to an arbitrary value of 1,000,000. Relative quantities are reported as the mean +/- standard deviation for each tissue.

Tissue	ApoF Ct	B-Actin	Relative Quantity
Brain	36.2	21.9	210 +/- 156
Heart	36.0	24.3	1815 +/- 2204
Lung	36.1	22.3	295 +/- 221
**Liver**	**25.5**	**23.7**	**1000000 +/- 467242**
Spleen	36.4	23.5	479 +/- 276
Kidney	36.2	24.8	3295 +/- 4758
Duodenum	38.0	22.3	94 +/- 98
Jejunum	39.1	22.0	54 +/- 75
Ileum	36.8	20.4	106 +/- 160
Inguinal Fat	36.3	24.9	1140 +/- 862
Brown Fat	37.1	26.0	1812 +/- 1259
Adrenals	31.5	22.1	5634 +/- 3241
Thyroid	36.2	22.8	328 +/- 115
Ovaries	33.5	21.6	1784 +/- 2450
